# Classification of Partial Discharge Signals by Combining Adaptive Local Iterative Filtering and Entropy Features

**DOI:** 10.3390/s18020406

**Published:** 2018-01-31

**Authors:** Imene Mitiche, Gordon Morison, Alan Nesbitt, Michael Hughes-Narborough, Brian G. Stewart, Philip Boreham

**Affiliations:** 1 Department of Engineering, Glasgow Caledonian University, 70 Cowcaddens Road, Glasgow G4 0BA, UK; imene.mitiche@gcu.ac.uk (I.M.); a.nesbitt@gcu.ac.uk (A.N.); mhnarborough@doble.com (M.H.-N.); 2 Institute of Energy and Environment, University of Strathclyde, 204 George Street, Glasgow G1 1XW, UK; brian.stewart.100@strath.ac.uk; 3 Innovation Centre for Online Systems, 7 Townsend Business Park, Bere Regis BH20 7LA, UK; pboreham@doble.com

**Keywords:** EMI method, partial discharge, permutation entropy, dispersion entropy, classification, expert’s system, EMI events (discharge sources)

## Abstract

Electromagnetic Interference (EMI) is a technique for capturing Partial Discharge (PD) signals in High-Voltage (HV) power plant apparatus. EMI signals can be non-stationary which makes their analysis difficult, particularly for pattern recognition applications. This paper elaborates upon a previously developed software condition-monitoring model for improved EMI events classification based on time-frequency signal decomposition and entropy features. The idea of the proposed method is to map multiple discharge source signals captured by EMI and labelled by experts, including PD, from the time domain to a feature space, which aids in the interpretation of subsequent fault information. Here, instead of using only one permutation entropy measure, a more robust measure, called Dispersion Entropy (DE), is added to the feature vector. Multi-Class Support Vector Machine (MCSVM) methods are utilized for classification of the different discharge sources. Results show an improved classification accuracy compared to previously proposed methods. This yields to a successful development of an expert’s knowledge-based intelligent system. Since this method is demonstrated to be successful with real field data, it brings the benefit of possible real-world application for EMI condition monitoring.

## 1. Introduction

Insulation condition monitoring is essential in High-Voltage (HV) equipment including insulated cables, transformers, large generators and motors which are operating in power plants. This allows early identification of several events related to insulation failure, such as Partial Discharge (PD) and Corona, which could lead to equipment breakdown followed by high maintenance or replacement costs. The process to identifying these insulation breakdown events can be performed through Electro-Magnetic Interference (EMI) diagnostics, which involves data sensing via the EMI technique and data analysis by experts. EMI diagnostics is a recognised technique used to identify the presence of insulation degradation and conductor-related faults in industrial HV machinery [1]. The downsides of an expert’s analysis are the high costs, human time and impracticability for continuous monitoring. In [2] the authors introduced, for the first time, an automatic and continuous condition monitoring solution, based on a pattern recognition approach. The developed model is seen as a transfer of expert knowledge to a software model. The classification results may be considered acceptable but with room for improved methodologies.

Currently, the most popular method for PD classification involves the analysis of Phase Resolved PD (PRPD) patterns [3,4]. To the author’s knowledge, the gaps found in the literature are as follows: (a) most of the proposed PD pattern recognition techniques are tested on simulated or experimental PD data (see [5,6,7,8,9,10,11]); (b) Techniques have addressed only the classification of different PD types; (c) classification using EMI data has not been fully analysed and is currently still under development.

In this paper, we improve the performance of previous EMI discharge sources classification [2,12,13,14,15], which aims to distinguish between a number of events including PD, Corona (C), Noise (N), Process Noise (PN) etc. Our previous method involves a state-of-the-art signal decomposition technique combined with an entropy measurement technique. Adaptive Local Iterative Filtering (ALIF) was used to decompose the EMI signal and obtain a number of time series signals at various frequency components, called Intrinsic Mode Functions (IMFs). This method has the benefit to effectively capture the amplitude and frequency variations in non-stationary signals compared to existing signal decomposition methods such as Empirical Mode Decomposition (EMD) [16]. PD signals are reported to be non-stationary [17], thus the obtained IMFs by ALIF may help to efficiently retrieve the unique time-frequency characteristics of each EMI event. Permutation Entropy (PE) is popular in biomedical application for electrocardiogram-type signals analysis and classification [18]. This type of signals exhibit the same behaviour as some of the captured EMI discharge signals, also referred to as events, and this is the main motivation for using the PE-based measure. The solution in this paper introduces a new feature extraction technique called Dispersion Entropy (DE), which is a modified and improved version of PE [19]. Both PE and DE are implemented to extract the features of each IMF signal, which are subsequently fed into a Multi-Class Support Vector Machine (MCSVM) classifier to distinguish between the different EMI events. The concept of these algorithms will be described later in this paper.

The next section briefly describes the overall process of the proposed solution. Section 3 describes in more detail the EMI monitoring approach used for signal measurement. Section 4 briefly defines the theory of the feature extraction and classification algorithms. Section 5 presents the application of the developed model to the measured data. Section 6 presents the classification results and finally conclusions are provided in the last section.

## 2. Proposed Solution

**Figure 1:**
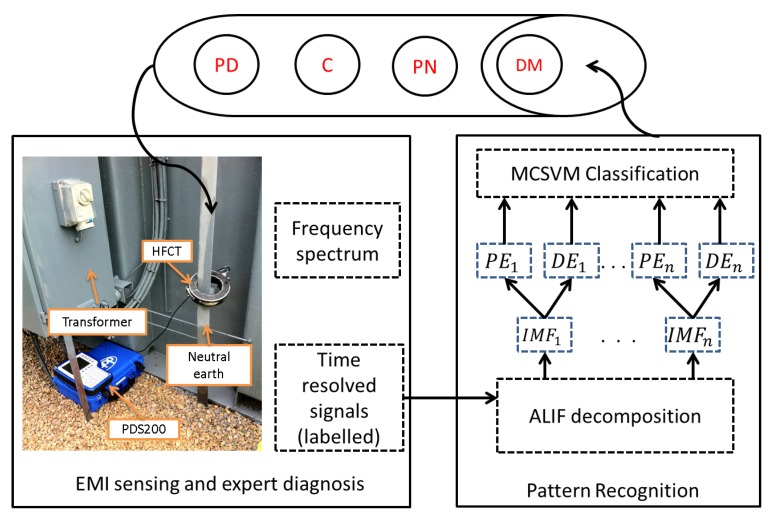
Overall process diagram of the proposed approach from data acquisition to pattern recognition of Electro-Magnetic Interference (EMI) events: Partial Discharge (PD), Corona (C), Process Noise (PN) and Data Modulation (DM).

Figure 1 outlines the method followed in the proposed classification solution. The data is first captured on power sites from various electrical apparatus. This figure illustrates an example of data sensed at a transformer. The data is captured using the EMI technique where a High Frequency Current Transformer (HFCT) is connected around the neutral earth cable. The data is logged into a device with quasi peak detection facility that provides a frequency spectrum from which the time resolved signals can be retrieved. Each signal is investigated by experts and subsequently labelled according to the event it contains (e.g., PD, C, N etc.) based on past experience of expert forensic confirmation. ALIF is used to decompose each signal into its multiple frequency components resulting in IMF time series from which PE and DE features are extracted. The feature vector is finally fed into an MCSVM classifier along with the relevant labels to train a model that can potentially learn and identify the characteristics of various events. Further details on EMI experimental setup and the measured data will be provided in Section 3 and Section 5 respectively.

## 3. EMI Measurement Technique

PD appears as a dielectric failure in electrical insulation apparatus due to HV stress; this results in an energy release that can produce EMI. EMI signals are well known to be generated by various types of faults in generators, motors, cables and associated auxiliary equipment [20]. The produced eruption of low energy electromagnetic pulses spreads in all directions from the discharge event. The emitted EMI signal can be sensed and analyzed across a wide frequency range. For instance, they can be sensed in the range as small as 10s of kHz while loose connections in an Isolated Phase Bus (IPB) can produce signals up to 100 MHz and above. EMI can both be radiated and conducted from the discharge site. The conducted energy is detected and measured with a split-core HFCT normally across a frequency range of 10 kHz to 100 MHz. The EMI measurement of these signals follows the CISPR-16-1-1 standard [21] to ensure compatibility of measurements and results interpretation across EMI measurement instruments. The split-core HFCT can be suitably located around the neutral connection conduit of a generator stator winding, at the grounding transformer, or, a motor cable supply conduit. The measurement of the EMI signals emitted is sensitive to various electrical and mechanical defects, including loose or broken stator and rotor bars, slot discharges, winding insulation defects, contamination on the windings, shaft eccentricity, bearing wear, etc. The severity, location and deterioration level of defects can often be measured by the EMI technique long before detection by means of more conventional methods. This is of value to applications that desire a system diagnostics approach. EMI analysis can distinguish between different defects and discharge sources, also referred to as events, and monitors not only activity within, e.g., a generator stator winding but, also, within adjacent auxiliary equipment as well. A number of issues have been identified by EMI techniques with exciters, cables, IPB and transformers, as well as machine stator windings. The resulting EMI spectrum is also unique for each physical location and type of defect within the electrical system [22].

## 4. Classification Theory

This section introduces the mathematical theory of ALIF, PE, DE feature extraction and MCSVM classification algorithms used in the proposed pattern recognition technique.

### 4.1. Adaptive Local Iterative Filtering

ALIF is a recursive approach that breaks down a multicomponent signal into a predefined number of IMFs, arranged from high to low frequency “space”, through subtraction of local averages from the signal [16]. The moving averages are calculated through convolution using low-pass filtering. Broadly, ALIF is a joining of EMD [23] with Iterative Filtering (IF) except that the filters are generated adaptively depending on the nature of the signal. For instance, if the signal’s characteristics change rapidly, short filters will be applied. On the contrary, if its characteristics change slowly, longer filters will be required. The filters are designed using Fokker–Planck equations in a Partial Differential Equation (PDE)-based model which is briefly explained in the following section. A complete and detailed explanation can be found in [16]. The mathematical theory of ALIF is described as follows. Let $f\left(n\right)=IMF_{1}\left(n\right)+IMF_{2}\left(n\right)+\ldots IMF_{K}\left(n\right)+R\left(n\right)$ be the multicomponent discrete signal which is decomposed into $IMF_{i}\left(n\right);i=1,2,\ldots ,K;K\in N$ IMFs plus a residual trend R(n). The coefficients of a low pass filter at step *j* and point *n* with length $2l_{j}\left(n\right)+1$ are denoted as: $h_{j}\left(n,\tau \right)\in R$, $\tau \in [-l_{j}\left(n\right),l_{j}\left(n\right)]$ where $l_{j}\left(n\right)$ is the variable that defines the filter’s length boundaries. The moving average of the signal calculated in each iteration is defined as:

(1) $$\;L_{j}\left(f\right)\left(n\right):=\sum_{\tau =-l_{j}\left(n\right)}^{l_{j}\left(n\right)}f\left(n+\tau \right)\cdot \;h_{j}\left(n,\tau \right).$$

The filtered output of ALIF is then obtained by Equation (2) from which the single IMF is extracted in what is called the inner-iteration.

(2) $$\;f_{j+1}\left(n\right)=f_{j}\left(n\right)-L_{j}\left(f\right)\left(n\right).$$

The algorithm uses a sequence of inner and outer iterations to obtain the IMFs. The inner iteration converges to save $f_{j}\left(n\right)$ as IMF. Starting with $f_{1}=f$, the operator $S_{1,j}\left(f_{j}\right)\left(n\right)=f_{j}\left(n\right)-L_{1,j}\left(f_{j}\right)\left(n\right)=f_{j+1}\left(n\right)$ captures the fluctuations in $f_{j}\left(n\right)$. Then, the first IMF is obtained as $IMF_{1}\left(n\right)=\lim_{n\to \infty }S_{1,j}\left(f_{j}\right)\left(n\right)$. In real implementations, *j* is limited according to a Stopping Distance (SD). For each step *j*, a threshold operator SD is estimated as follows:

(3) $$\;D=\frac{||IMF_{1,j}-IMF_{1,j-1}{\left|\right|}_{2}}{||IMF_{1,j-1}{\left|\right|}_{2}}.$$

The inner iteration is stopped when the *D* value reaches a particular threshold as recommended in [23,24]. The outer iteration converges when the number of local extreme points in $f_{j}\left(n\right)$ is at most one, in other words, when the signal is a trend. This results in the residual, calculated as $R\left(n\right)=f-IMF_{1}-\ldots -IMF_{K-1}$. In practice, another criterion is added to stop the outer iteration which is when the number of desired IMFs is reached.

### 4.2. Permutation Entropy

**Figure 2:**
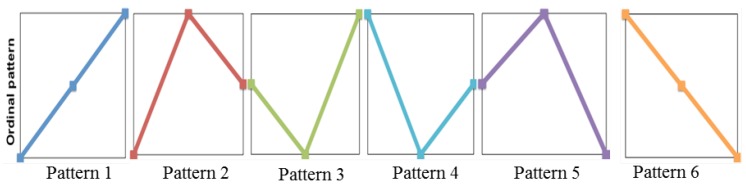
Permutation Entropy (PE) ordinal patterns for m=3.

**Figure 3:**
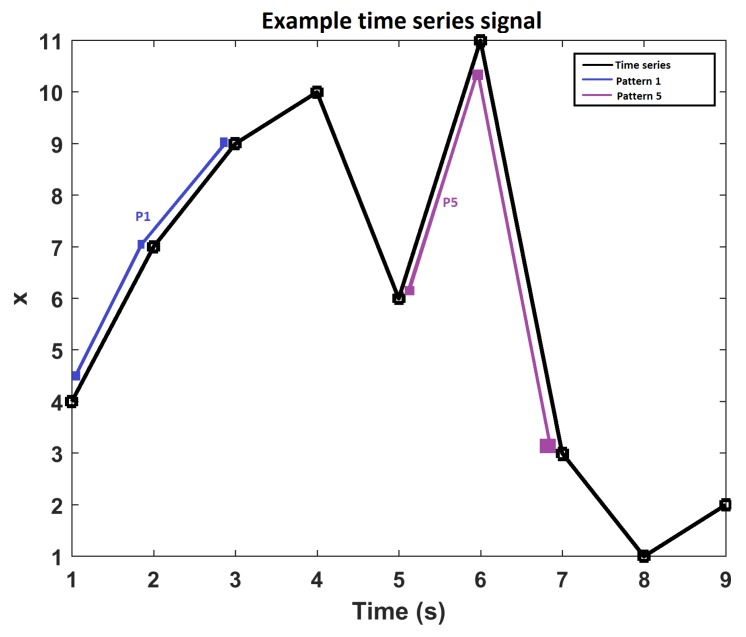
Example of pattern mapping/identification in a time series.

PE was developed by Bandt and Pompe [25] to assess the complexity in time series data with more resilience to low frequency artefacts. This makes it suitable for measuring real-world, noisy and chaotic time series signals. PE is derived from Shannon’s entropy theorem which is a measure of information contained in a data set [26]. Bandt and Pompe combined the entropy concept with symbolic dynamics to create a simple, fast to compute, robust and stable measure of regularity in short time series and to overcome classical entropy method limitations, including the requirement of long data sets and high computational cost [27]. Figure 2 illustrates m!=6 possible ordinal patterns, for an embedded dimension of m=3, that can be mapped to a time sequence data set. Figure 3 demonstrates pattern identification in an example time series, where instances of pattern 1 and 5 are plotted. Note that the time series from 3 to 5 s would be identified as pattern 5 and the time series from 7 to 9 s would be identified as pattern 4. The frequencies of pattern occurrences across the complete time series signal are calculated and presented in a histogram which is normalised in order to obtain the permutation probabilities that are used to compute the PE value.

The PE mathematical theory is described in [28] as follows. First, we denote vectors and scalars by upper and lower case respectively. Based on a given time series $\left\{x_{1},x_{2},\ldots ,x_{N}\right\}$, vectors X(j); j=1,2,…,N−(m−1) are constructed as:

(4) X(j)={x(j),x(j+1),…,x(j+(m−1))}.

The sequence in Equation (4) is arranged to provide components in increasing order as follows:

(5) $$\;\{x\left(j+\left(i_{1}-1\right)\right)\leq x\left(j+\left(i_{2}-1\right)\right)\leq \ldots \leq x\left(j+\left(i_{m}-1\right)\right)\}.$$

If two successive components are equal, i.e., $x\left(j+\left(i_{1}\right)\right)=x\left(j+\left(i_{2}\right)\right)$ for $i_{1}\leq i_{2}$, then their positions can be rearranged to $x\left(j+\left(i_{1}\right)\right)\leq x\left(j+\left(i_{2}\right)\right)$. Next, a different symbol series S(l) is calculated for each time series x(i) as:

(6) $$\;S\left(l\right)=\left(i_{1},i_{2},\ldots ,i_{m}\right)$$

where l=1,2,…,k(k=m!). That is, there will be m! different symbol series or permutations $\pi_{n}$. The probability $p(\pi_{n}^{m})$ of each symbol sequence S(l) is then calculated mathematically as:

(7) $$\;p\left(\pi_{n}^{m}\right)=\frac{\sum_{j\leq N}^{1}u:type\left(u\right)=\pi_{n}\left(X_{n}^{m}\right)}{\sum_{j\leq N}^{1}u:type\left(u\right)\in \Pi \left(X_{n}^{m}\right)}.$$

The mapping of the symbol S(l) to the ordinal pattern is denoted as type(.) and the m! symbols ${\left\{\pi_{n}^{m}\right\}}_{n}^{m!}$ are denoted as Π. The indicator function $1_{A}\left(u\right)$ of a set *A* is defined as:

$$\;1_{A}\left(u\right)=\left\{\begin{array}{c} 1\;if\;u\in A\\ 0\;if\;u\notin A \end{array}\right.$$

Finally, the value of *PE* can be estimated using the formula:

(8) $$\;PE\left(m\right)=-\sum_{n=1}^{m!}p\left(\pi_{n}^{m}\right)\cdot log\left(p\left(\pi_{n}^{m}\right)\right).$$

PE should be suitable to quantify the characteristics contained in EMI signals since they are by nature non-stationary and complex to analyse.

### 4.3. Dispersion Entropy

DE was introduced in [19] to overcome (PE) and Sample Entropy (SE) limitations. SE is slow in computation particularly for long time series, whereas PE disregards information of the amplitude values mean and amplitude variations [29]. DE is calculated as follows. Given a time series signal X$=\{x_{1},x_{2},\ldots ,x_{N}\}$, with length of *N*, let X be mapped to Y$=\{y_{1},y_{2},\ldots ,y_{N}\}$ using the Normal Cumulative Distribution Function (NCDF). Next, each $y_{j};j=1,\ldots ,N$ is assigned a class from 1 to *c* linearly as follows.

(9) $$\;z_{j}^{c}=round\left(c\cdot y_{j}+0.5\right).$$

This provides *N* members of the classified time series. Here, other linear or non-linear methods can also be employed. Next, embedding vectors $Z_{i}^{m,c}$ with dimension *m* and time delay *d* are created:

(10) $$\;Z_{i}^{m,c}=\left\{z_{i}^{c},z_{i+d}^{c},\ldots ,z_{i+(m-1)d}^{c}\right\};\;i=1,2,\ldots ,N-\left(m-1\right)d.$$

The latter is mapped to a dispersion pattern $\pi_{v_{o}v_{1}\ldots v_{m-1}}$, among $c^{m}$ possible dispersion patterns, in that $v_{0}=z_{i}^{c},v_{1}=z_{i+d}^{c},\ldots ,v_{m-1}=z_{i+(m-1)d}^{c}$.

The dispersion probability of occurrence for each pattern is then calculated as follows.

(11) $$p\left(\pi_{v_{o}v_{1}\ldots v_{m-1}}\right)=\frac{\sum_{i\leq N-(m-1)d}1_{u:type\left(u\right)=\pi_{v_{o}v_{1}\ldots v_{m-1}}}\left(Z_{i}^{m,c}\right)}{N-(m-1)d}.$$

Finally, the *DE* value is obtained based on the Shannon entropy formula as follows.

(12) $$\;DE\left(m,c,d\right)=-\sum_{1}^{c^{m}}p\left(\pi_{v_{o}v_{1}\ldots v_{m-1}}\right)\cdot log\left(p\left(\pi_{v_{o}v_{1}\ldots v_{m-1}}\right)\right).$$

The calculated *DE* value measures the spreading in a time series. This information may be useful to obtain the IMF characteristics.

### 4.4. Support Vector Machine

SVM is a regression and binary classification technique which was introduced in [30]. The algorithm aims to locate a hyperplane that separates two groups of data features. This method is used in various fields and in a wide range of different applications including text classification, sound recognition, image categorisation, and data classification [31]. SVM is popular for its high-dimensional features implementation while providing high detection accuracy compared to other classification algorithms such as neural networks and random forests [32]. This benefits fault classification by reducing confusion. The separation process of a data set belonging to two different classes is achieved in two main steps:

1. SVM generates an optimal line that separates the two different data features, such that feature clusters of one class are grouped on one side of the feature space and the remaining ones are grouped on the other side. This yields to the SVM model which is used in future data classification.
2. SVM classifies the training data set based on the trained model in the previous step; this is known as the testing phase.

**Figure 4:**
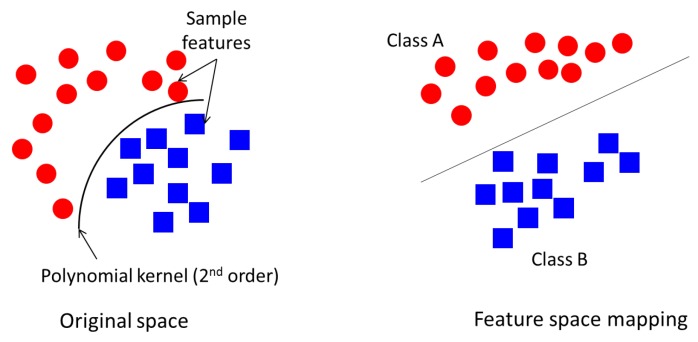
Support Vector Machine (SVM) linear space mapping using second-order polynomial kernel function.

The distance between the hyperplane and the nearest point of each class is called the margin. The wider the margin, the better the separation between classes. The separation is achieved by a kernel function such as linear, quadratic, radial basis or polynomial function. For instance, a polynomial function seeks to draw a non-linear line to first separate the data, then map the data to a linear space as illustrated in Figure 4.

The choice of kernel function implementation depends on the nature of the data. The classic approach to find the suitable kernel function and its parameters for optimum performance is a Grid Search method. This involves multiple training/testing of the MCSVM with all possible kernel functions and their parameters [33]. The mathematical theory of SVM is described as follows. Let $x_{i}$ be the data input and $y_{i}$ the associated labels in that i=1,2,…,L, where *L* is the number of data samples. It is assumed that the data points belong to two classes “A” and “B”. Each data point is non-linearly mapped to a feature space separated by a hyperplane with the basic geometric equation:

(13) f(x)=w×x+b=0

where *b* is a scalar and *w* is a P-dimensional vector. These are the key parameters that determine the hyperplane position. If b=0, the hyperplane will pass by the origin. Otherwise, the margin is created or increased. The parallel hyperplanes that separate the two different data classes are defined in Equation (14) for the first class and in Equation (15) for the second one.

(14) w×x+b=1.

(15) w×x+b=−1.

Through geometric calculations, the distance between the hyperplanes or the margin width is $\frac{2}{\left|w\right|}$. This maximises the margin width to achieve an optimum separation between the two classes. In order to maximise the margin width, |w| should be minimised which brings in the criteria: $w\times x_{i}+b\geq 1$ for the first class or $w\times x_{i}+b\geq -1$ for the second one. This will force the points from each class not to exceed the class hyperplanes. The samples located on the hyperplanes are named support vectors and this is from where the name SVM originates. The hyperplane is obtained as a solution to the optimisation problem in Equation (16), while considering the noise slack variable $\zeta_{i}$ which determines the range to which the samples overstep the margin, and the error penalty *C* which represents the trade-off between maximisation of the margin and classification error during the training phase.

(16) $$\;min\frac{1}{2}{\left||w|\right|}^{2}+C\sum_{i=1}^{M}\zeta_{i}$$

$$Subject\;to\left\{\begin{array}{cc} y_{i}\left(w^{T}\times x_{i}+b\right)\geq 1-\zeta_{i}\\ \zeta_{i}\geq 0, & i=1,\ldots ,M \end{array}\right.$$

where $\zeta_{i}$ denotes the distance between the margin and the data point $x_{i}$ which is in error. The calculation of Equation (16) is simplified and solved through a Lagrangian problem which is explained in more detail in [34]. This introduces an $\alpha_{i}$ parameter which expresses *w* in solving Equation (16). The solution yields to a non-linear decision function expressed as:

(17) $$\;f\left(x\right)=sign(\sum_{i,j=1}^{M}\alpha_{i}y_{i}\left(x_{i}x_{j}\right)+b).$$

The challenges that may be faced in the SVM learning process of high dimension feature space are data over-fitting and computational errors. Over-fitting can be solved by introducing a kernel function which performs a dot product of the feature space, i.e., $K\left(x_{i},x_{j}\right)=\left(\Phi^{T}\left(x_{i}\right)\times \Phi \left(x_{j}\right)\right)$. The definition of this kernel function can be found in [30] . A non-linear vector function $\Phi \left(x\right)=(\phi_{1}\left(x\right),\ldots ,\phi_{l}\left(x\right))$, where *l* is the feature space’s dimension, is implemented to reformulate the decision function in Equation (17) to:

(18) $$\;f\left(x\right)=sign(\sum_{i,j=1}^{M}\alpha_{i}y_{i}\left(\Phi^{T}\left(x_{i}\right)\times \Phi \left(x_{j}\right)\right)+b).$$

Since SVM is a binary classifier, it cannot handle more than two classes. This is not suitable for classification of multiple discharge sources. For this reason, MCSVM is implemented using the One-Against-One approach where k(k−1)/2 models are constructed. Each model is trained on two classes, *A* and *B*, as a normal binary classification. The testing step is performed through a “Max Win” voting method. If the closest class to the test sample is class *A*, then the vote for this class is increased by one. The winning class is the one which has the highest number of votes.

## 5. Application to EMI Data

The data was sensed while the assets were operating using the described EMI method in Section 3. The time-resolved signals were recorded at a sampling rate of 24 kHz by means of a PD Surveyor 200 (PDS200) device which follows the CISPR-16-1-1 standard for EMC type filtering. The PDS200 is used for PD surveying and has the ability to detect and analyse Radio Frequency Interference (RFI) as well as EMI radiation. PDS200 is identical to PDS100 except that it has a lower frequency range that starts from 50 kHz up to 1 GHz. In contrast, PDS100 operates in the range of 50 MHz–1 GHz. The low frequency option makes the PDS200 suitable to use for EMI detection and analysis, unlike PDS100 which is used to detect RFI emission, in the Ultra-High-Frequency (UHF) range, only for RFI surveying by means of antennas [35]. It also provides a frequency spectrum of the recorded signals using the EMI standard quasi peak detection method. “EMI experts” selected the time signals measured at a frequency of interest in the spectrum for further analysis and event identification, then they labelled the event type in each signal. These labels help in training the classification algorithm. Some of these signals were found to contain more than one event and therefore were given a combination of two labels (e.g., PD+A) which is considered as a single label. In this work, the multi-labelled signals are considered as a single class. Each measured signal contains 500 cycles over 10s. The overall data was collected from three different sites which are described as follows.

1. Site 1: The data was measured at the neutral earth cable of a 661 MVA hydrogen/water cooled synchronous generator operating at 23.5 kV, 19 kA, 3 phase, 50 Hz, 3000 RPM, 0.85 lag/0.95 lead power factor and 2 pole. A total of 13 signals were identified to contain E+mPD, C+E, C, N, PN and mPD.
2. Site 2: similar to the previous site, the measurements were taken at the neutral earth of different assets including a General Step-Up (GSU) Transformer operating at 430/15.5 kV, 444/12329 A, 3 phase, 50 Hz, 331 MVA, IPB and Station Transformer (Sta XFMR). The events identified in the GSU transformer are mPD+mA, PD+mA, PD+A, PD and the events identified in the IPB are PD and PN. Finally, the DM event was identified in the Sta XFMR.
3. Site 3: The data was measured at the neutral earth cable of an H2 cooled generator, operating at 294.25 MVA, 15 kV, 0.85 PF, 2 pole and 3000 RPM, from which seven signals were selected with an additional signal selected from an H2 cooled Steam Turbine Generator (STG) operating at 15 kV, 2 pole and 3000 RPM. The labelled events found at the neutral earth cable are PN, PD, NVFD, E and the ones found at the STG are: PD, E+mPD and E+PD.

A fourth scenario of common events between sites is also considered. This data set is a subset of the data collected across the three sites and includes PN, PD and mPD. Note that “m”, stands for a minor event which implies an occurrence at a smaller rate and lower discharge level. Here, mPD is considered as a type of PD, thus it is worth investigating its classification among PD signals observed in the other sites.

**Figure 5:**
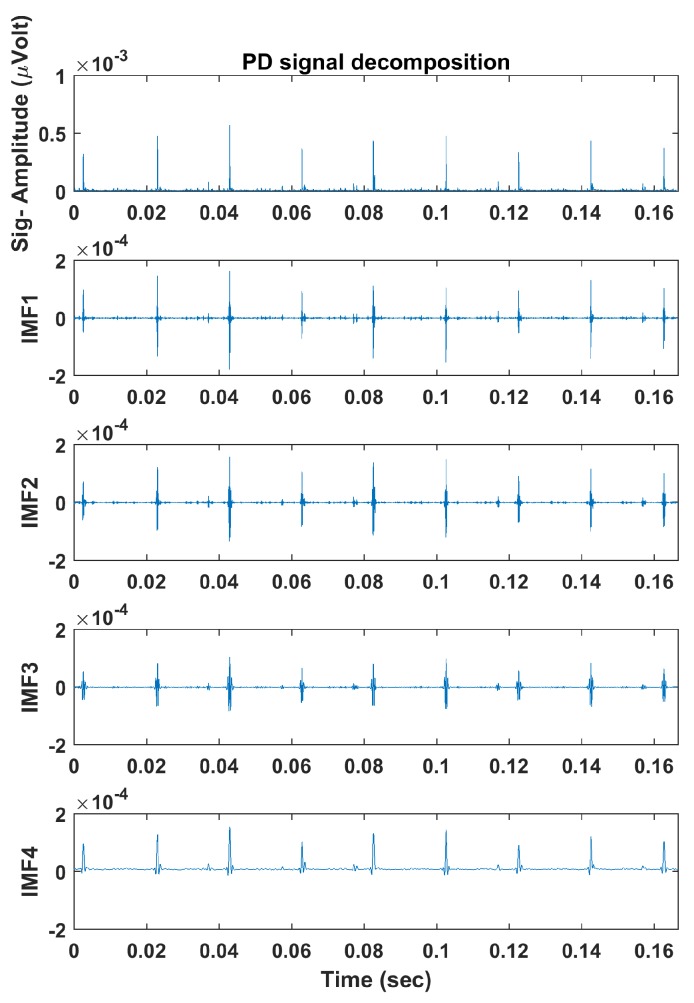
Example PD signal decomposed into Intrinsic Mode Functions (IMFs) using the Adaptive Local Iterative Filtering (ALIF) algorithm.

**Figure 6:**
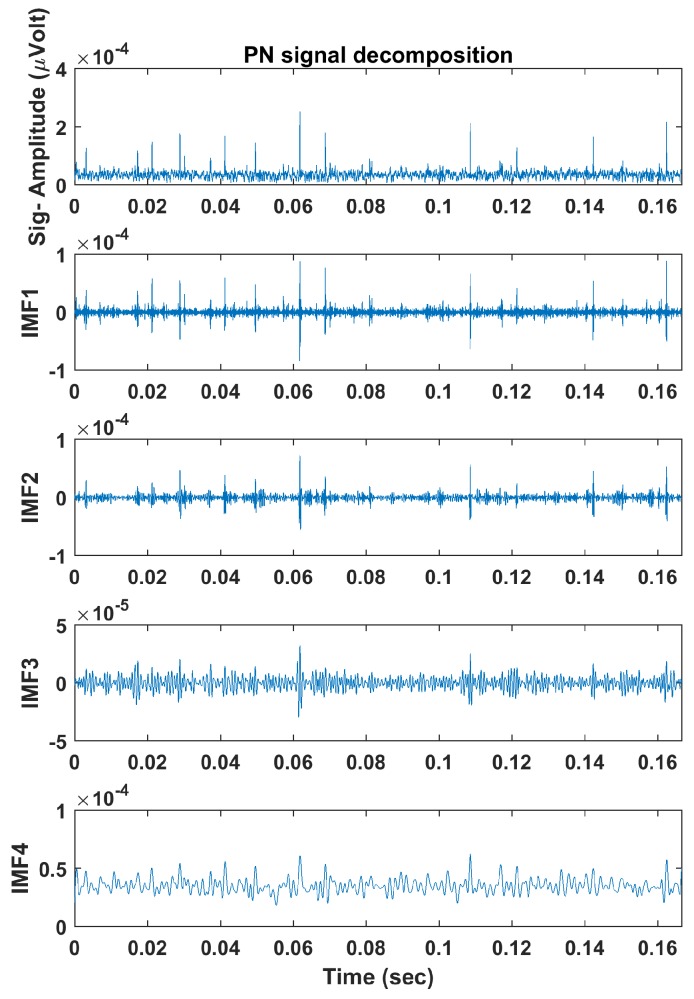
Example PN signal decomposed into IMFs using the ALIF algorithm.

The described algorithms in Section 4 were applied, and each signal’s multiple frequency components were obtained using ALIF. Figure 5 and Figure 6 illustrate example PD and PN signals respectively and their decomposed IMFs using ALIF. Next, PE and DE values were calculated, with m=3 and d=1, for each IMF to obtain a 1 × 8 feature vector for each signal. Table 1 presents the PE and DE values calculated for each IMF of the illustrated signals in Figure 5 and Figure 6. Differences in IMFs as well as their PE and DE values are observed between PD and PN signals, thus successful classification should be expected. The feature vectors are finally implemented in MCSVM which would draw boundaries that group the IMFs’ PE and DE values in the feature space. Polynomial kernel functions of second- and third-order were implemented based on the findings of a Grid Search method [33]. The training/testing strategy followed a ten-fold hold-on cross validation method, where the model is trained on 90% of the data and is tested on the remaining 10% in ten iterations; each uses a random different data batch. The total classification accuracy is calculated as the average accuracy resulting from all iterations. Training of SVM implies presenting the training data set and its associated labels to the classifier. In the testing phase the unseen data set is presented to the classifier, which will subsequently predict the data labels. The predicted labels are compared to the true labels that are provided by “EMI experts”, and then the accuracy % is calculated. A potential limitation of the work is that the labels may not be accurate, yet a small number of mislabelled data may occur. However, an experienced expert with modest training is capable of identifying many of the common faults related to EMI signals. Furthermore, one mislabelled instance is not likely to greatly affect the classification accuracy as the SVM algorithm would draw a boundary around the majority of instances belonging to a particular class. The classification process is performed using all the data in each scenario (site 1, 2, 3 and common events between sites) discussed previously.

## 6. Results and Discussion

**Figure 7:**
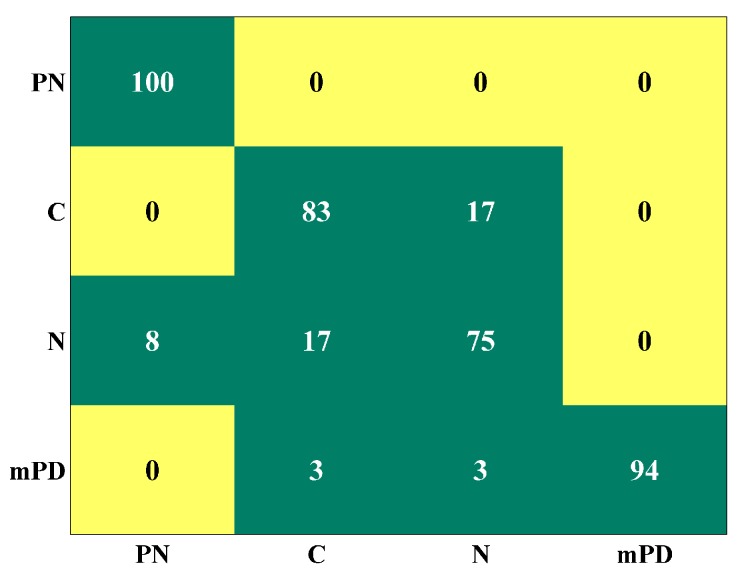
Confusion matrix of site 1.

Classification accuracy for each case is illustrated in Table 2. Remarkably, 100% classification accuracy is observed in sites 2, 3 and in the common data subset scenario. Lower classification accuracy is observed in site 1. However, it is clear that an improvement in accuracy is achieved for each case, compared to [2]. An improvement of 8% is achieved for site 1 and 3 and an improvement of 14% and 4% is achieved for sites 2 and the common condition case respectively. In order to further explain the performance of the ALIF-PE, DE algorithm in site 1, the confusion matrix between event classifications is presented in Figure 7. This matrix shows the class (columns) in which an event (rows) was classified. The highest confusion (17%) is observed between C and N. This could be due to the presence of noise that has similar characteristics to N in the C signal as noise is almost inevitable in real-world signals, especially in an electrical environment. A confusion of 8% between N and PN is also observed. mPD has minor confusion with C and N of 3% each. The overall findings lead to the conclusion that EMI events can be distinguished regardless of the site or origin of the asset’s collected data. It is important to highlight that classification accuracy may be affected if more events or more data are implemented in the classifier.

## 7. Conclusions

This work introduces an extended pattern recognition solution for real-world EMI signal classification. The solution involves extraction of relevant time and frequency fingerprints using ALIF plus PE and DE entropy-based features. MCSVM was also used to classify different EMI signals collected in three power system sites. Results demonstrate an improved classification accuracy over the previously proposed approach. The other significant contribution is the successful classification of common sources between different sites. The outcome of this work could potentially be exploited to develop a condition monitoring software which is based on EMI expert system knowledge. 

## Figures and Tables

**Table 1:** PE and Dispersion Entropy (DE) values for each IMF of the example PD and PN signals.

Signal	Feature	IMF1	IMF2	IMF3	IMF4
**PD**					
	PE	1.75	1.45	1.24	1.06
	DE	1.13	1.42	1.50	1.34
**PN**					
	PE	1.79	1.39	1.14	0.95
	DE	2.01	1.82	1.64	1.39

**Table 2:** Classification accuracy results.

Case	Classification Accuracy %
Site 1	91
Site 2	100
Site 3	100
Common data subset	100

## Abbreviations

The following abbreviations are used in this manuscript:

A	Arcing
ALIF	Adaptive Local Iterative Filtering
C	Corona
DE	Dispersion Entropy
DM	Data Modulation
E	Exciter
EMI	Electro-Magnetic Interference
GSU	General Step-Up
HFCT	High Frequency Current Transformer
HV	High Voltage
IMF	Intrinsic Mode Function
IPB	Isolated Phase Bus
MCSVM	Multi-Class SVM
NVFD	Non Variable Frequency Drive
PD	Partial Discharge
PDE	Partial Differential Equation
PDS200	Partial Discharge Surveyor 200
PE	Permutation Entropy
PN	Process Noise
PRPD	Phase Resolved PD
SD	Stopping Distance
STG	Step Up Generator
Sta XFMR	Station Transformer
SVM	Support Vector Machine
UHF	Ultra-High-Frequency
